# CD157 selectively identifies hPSCs with enhanced hepatic differentiation capacity

**DOI:** 10.1093/lifemedi/lnae026

**Published:** 2024-06-05

**Authors:** Shuang Li, Dacheng Jiang, Xin Li, Yongxu Zhao, Xiaosong Gu, Chunping Jiang, Qiurong Ding

**Affiliations:** CAS Key Laboratory of Nutrition, Metabolism and Food Safety, Shanghai Institute of Nutrition and Health, Shanghai Institutes for Biological Sciences, University of Chinese Academy of Sciences, Chinese Academy of Sciences, Shanghai 200031, China; Center for Metabolic Disease Drug Research, Shandong Laboratory of Yantai Drug Discovery, Bohai Rim Advanced Research Institute for Drug Discovery, Yantai 264117, China; CAS Key Laboratory of Nutrition, Metabolism and Food Safety, Shanghai Institute of Nutrition and Health, Shanghai Institutes for Biological Sciences, University of Chinese Academy of Sciences, Chinese Academy of Sciences, Shanghai 200031, China; Regenerative Medicine and Tissue Engineering Research Platform, Jinan Microecological Biomedicine Shandong Laboratory, Jinan 250021, China; Center for Metabolic Disease Drug Research, Shandong Laboratory of Yantai Drug Discovery, Bohai Rim Advanced Research Institute for Drug Discovery, Yantai 264117, China; Regenerative Medicine and Tissue Engineering Research Platform, Jinan Microecological Biomedicine Shandong Laboratory, Jinan 250021, China; Regenerative Medicine and Tissue Engineering Research Platform, Jinan Microecological Biomedicine Shandong Laboratory, Jinan 250021, China; Department of Hepatobiliary Surgery, The Affiliated Drum Tower Hospital of Nanjing University Medical School, Nanjing 210093, China; CAS Key Laboratory of Nutrition, Metabolism and Food Safety, Shanghai Institute of Nutrition and Health, Shanghai Institutes for Biological Sciences, University of Chinese Academy of Sciences, Chinese Academy of Sciences, Shanghai 200031, China

**Dear Editor,**

Cellular metabolism has been recognized as a critical regulator of pluripotency in human pluripotent stem cells (hPSCs) [1, 2]. Several studies have reported genetic, epigenetic, and transcriptional variations among hPSC cultures [3, 4]. In our study, we aimed to investigate the potential metabolic heterogeneity in hPSCs. To do so, we introduced genetic probes, including MitoTimer (indicating mitochondrial oxidative stress), Grx (reflecting the ratio of GSH/GSSG), Trx (representing the ratio of NADPH/NADP^+^), and SoNar (measuring the ratio of NAD^+^/NADH), into a human pluripotent stem cell line (1016) using lentiviral vectors (Fig. S1A). While we observed some differences with the Grx and Trx probes, we noticed the most significant heterogeneity in the NAD^+^/NADH ratio among cells, as revealed by the SoNar probe (Fig. 1A). The heterogeneity of the NAD^+^/NADH ratio was also observed in another two hPSC lines, although to a less extend (Fig. S1B). We subsequently sorted out the top 4% cell population (SoNar-High, displaying high NAD^+^/NADH) and the bottom 4% cell population (SoNar-Low, exhibiting low NAD^+^/NADH) from the 1016 hPSCs. Interestingly, after several independent passages of these two populations, they maintained the relative differences in the NAD^+^/NADH ratio, as demonstrated by the SoNar probe (Figs. 1B and S2A), as well as through direct measurement of NAD^+^ and NADH levels in cells (Fig. 1C). These findings suggest that the heterogeneity of the NAD^+^/NADH ratio arises from intrinsic variations in cells that result in differences in NAD metabolism.

**Figure 1.**
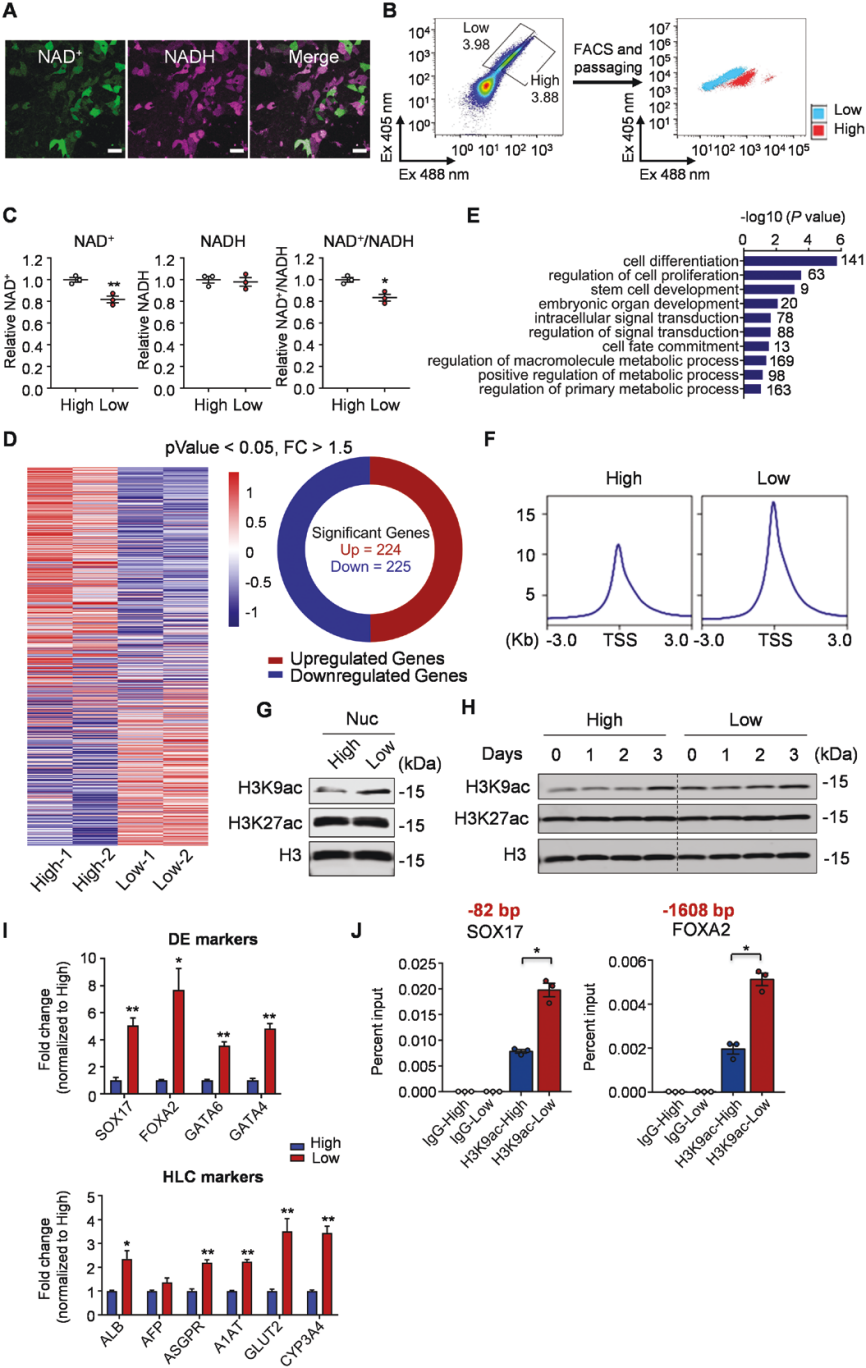
The cellular heterogeneity of NAD^+^ level in hPSCs affects the endoderm and hepatic differentiation capacity. (A) Representative images for the NAD^+^/NADH fluorescence in 1016 hPSCs infected with SoNar lentivirus. Scale bar, 50 µm. (B) hPSCs infected with SoNar lentivirus were sorted by SoNar probe to obtain SoNar-High (NAD^+^/NADH high) and SoNar-Low (NAD^+^/NADH low) cells. A diagram of cells with high and low NAD^+^/NADH ratio sorted by around 4% ratio using SoNar probe (left). Analysis of cells sorted out with high and low NAD^+^/NADH ratios after multiple generations (right). (C) Measurement of the NAD^+^/NADH ratio in SoNar-High and -Low cells, respectively. *n* = 3 biological replicates. (D, E) Heatmap presentation (D) and pathway analysis (E) of differentially expressed genes in SoNar-High and -Low cells. (F) Average ATAC-seq signals in SoNar-High and -Low cells. (G) Western analysis of the H3K9ac and H3K27ac signals in the SoNar-High and -Low hPSC. Nuc, nuclear. (H) Western analysis of the H3K9ac and H3K27ac signals in the SoNar-High and -Low hPSC during definitive endoderm (DE) differentiation. (I) Relative mRNA levels of DE and hepatocyte-like cell (HLC) marker genes in SoNar-High and -Low hPSCs-derived DE cells (above) and HLCs (below), respectively. *n* = 3 biological replicates. (J) Chromatin immunoprecipitation (ChIP) analysis with H3K9ac antibody in SoNar-High and -Low cells-derived DEs. The 82 bp and 1608 bp indicate the upstream position of ChIP-qPCR primers from the transcription start site. *n* = 3 biological replicates. The results are presented as means ± SEM; *P < 0.05; **P < 0.01. Two-tailed Student’s *t* test was used for two-group comparisons, and one-way ANOVA was used for multi-group comparisons

NAD^+^ plays a vital role as a coenzyme in energy metabolism and serves as a rate-limiting co-substrate for various enzymes, including the deacetylase sirtuins [5]. We next compared these two populations (SoNar-High *vs.* SoNar-Low) based on their epigenetic and transcriptional profile, as well as pluripotency. Our RNA-seq analysis revealed significant differences in gene expression between the two groups. We identified 224 significantly upregulated genes and 225 significantly downregulated genes in the SoNar-High group compared to the SoNar-Low group (Fig. 1D). These differentially expressed genes were primarily enriched in pathways related to cell differentiation, cell proliferation, and others (Fig. 1E). Additionally, our ATAC-seq analysis demonstrated that the SoNar-Low group exhibited significantly more signals, suggesting increased chromatin accessibility (Fig. 1F). Consistently, the SoNar-Low group showed enhanced signal in nuclear H3K9ac (Fig. 1G), which could be attributed to lower sirtuin activity compared to the SoNar-High group.

Notably, we found that cells in the SoNar-Low group have enhanced clonal formation capacity, suggesting an increased ability for self-renewal (Fig. S2B). Although RNA-seq analysis showed no significant change in expression levels of most transcription factors involved in regulating PSC’s stemness (Fig. S2C), the SoNar-Low cells displayed higher efficiency in differentiating into endoderm and hepatocytes, as well as mesoderm (Figs. S2D–F, 1I). However, their differentiation into ectoderm neuronal cells appeared to be impaired (Fig. S2G). In line with these findings, we observed an increased signal of H3K9ac in the SoNar-Low cells when subjected to endoderm differentiation, both in cell lysates (Fig. 1H) and the promoter regions of *SOX17* and *FOXA2* genes, which are typical markers of endoderm cells (Fig. 1J). Collectively, these results provide clear evidence of variations in pluripotency, as well as epigenetic and transcriptional profiles, between these two populations characterized by different levels of NAD metabolism.

To investigate the underlying causes of the differences in NAD metabolism between the SoNar-High and SoNar-Low populations, we aimed to explore changes in DNA methylation, as long-term *in vitro* culture of hPSCs has been associated with the accumulation of DNA methylation aberrations that can be inherited to daughter cells [3, 4]. We conducted whole-genome bisulfite sequencing (WGBS-seq) to examine DNA methylation changes between two groups. We identified differences in DNA methylation regions near a total of 86 genes, with increased methylation signals near 24 genes and decreased signals near 62 genes, specifically in the SoNar-Low population. Interestingly, these 86 genes were functionally enriched in the regulation of cellular biosynthetic and metabolic processes (Fig. S3A). Notably, several genes involved in the biosynthetic process, such as *SOX10* (SRY box transcription factor 10), *MESP1* (Mesoderm Posterior BHLH transcription factor 1), and several members in the zinc finger family, exhibited changes in DNA methylation (Fig. S3B). We then performed an overlap analysis with the previous ATAC-seq and RNA-seq results, which revealed genes that consistently showed changes in all three high-throughput profiling assays. This suggests the existence of an epigenetic-transcriptional axis involving these genes between the SoNar-High and SoNar-Low cells (Fig. S3C and S3D). Of particular interest are two genes, *ART3* (ADP-ribosyl transferase 3) and *BST1* (bone marrow stromal cell antigen 1), as they encode proteins that serve as surface markers and are functionally involved in cellular NAD metabolism [6, 7] (Fig. S3C).

The *ART3* gene encodes an arginine-specific ADP-ribosyltransferase that catalyzes a reversible reaction involving the addition or removal of ADP-ribose to an arginine residue on target proteins, thereby regulating their function. The enzymatic reaction utilizes NAD^+^ as a substrate. In our study, we observed decreased DNA methylation signals in a specific region of the *ART3* gene in the SoNar-Low population. This was accompanied by significantly increased ATAC-seq signals, and enhanced expression of *ART3* (but not *ART5,* the other member in this family), in the SoNar-Low cells (Fig. S4A and S4B). Interestingly, when we used CRISPR technology to deplete *ART3* in hPSCs (Fig. S4C), we observed a significant increase in NAD^+^ levels, as well as an elevated ratio of NAD^+^/NADH (Fig. S4D). Conversely, overexpression of *ART3* in hPSCs led to a decrease in NAD^+^ levels and the ratio of NAD^+^/NADH (Fig. S4E). These results collectively suggest that the methylation and expression of ART3 may contribute to the heterogeneity of NAD metabolism in hPSCs. However, we encountered challenges in identifying commercially available ART3 antibodies that can reliably sort out hPSCs based on ART3 expression level. Further investigations are needed to explore whether ART3 can serve as a surface marker indicative of NAD metabolism in hPSCs.

We next examined the role of BST1 in our study. BST1, also known as CD157, belongs to the ADP-ribosyl cyclase gene family. Previous research has identified CD157 as a marker of tissue-resident vascular endothelial stem cells with strong self-renewal capacity and vascular regeneration potential [8]. In our investigation, we observed a decreased DNA methylation signal in a specific region of the *BST1* gene in the SoNar-Low population (Fig. 2A), along with an increased ATAC-seq signal (Fig. 2A) and elevated expression of *BST1* (Fig. 2B). Although manipulating BST1 expression using CRISPR technology or overexpression did not impact cellular NAD^+^ levels (Fig. S5), we successfully identified an antibody that specifically labels surface CD157, which exhibited heterogeneous expression in hPSCs (Fig. 2C). To further investigate the functional significance of CD157, we sorted hPSC subpopulations based on surface CD157 levels. Consistent with our previous findings, the CD157-high population displayed a lower NAD^+^ level and a decreased NAD^+^/NADH ratio, while the CD157-low population exhibited the opposite trend (Fig. 2D). Interestingly, the CD157-high population demonstrated a significant enhancement in endoderm and hepatocyte differentiation capacity, as evidenced by increased expression levels of cell identity markers (Fig. 2E, 2F, and 2H) and enhanced albumin secretion (Fig. 2G). RNA-seq analysis of hepatocytes derived from these two populations revealed significant differences in genes involved in metabolic pathways, cholesterol metabolism, and other processes (Fig. 2I). Furthermore, analysis of liver tissue-enriched genes from the Human Protein Atlas project [9] (Human Protein Atlas proteinatlas.org) revealed 112 genes exhibiting significant differential expression (Fold change > 1.5, *P* < 0.05) between the CD157-high and CD157-low populations. The majority of these 112 genes showed significantly higher expression in CD157-high hPSC-derived hepatocytes (Fig. 2J), indicating a greater efficiency of hepatic differentiation in the CD157-high hPSC population.

**Figure 2.**
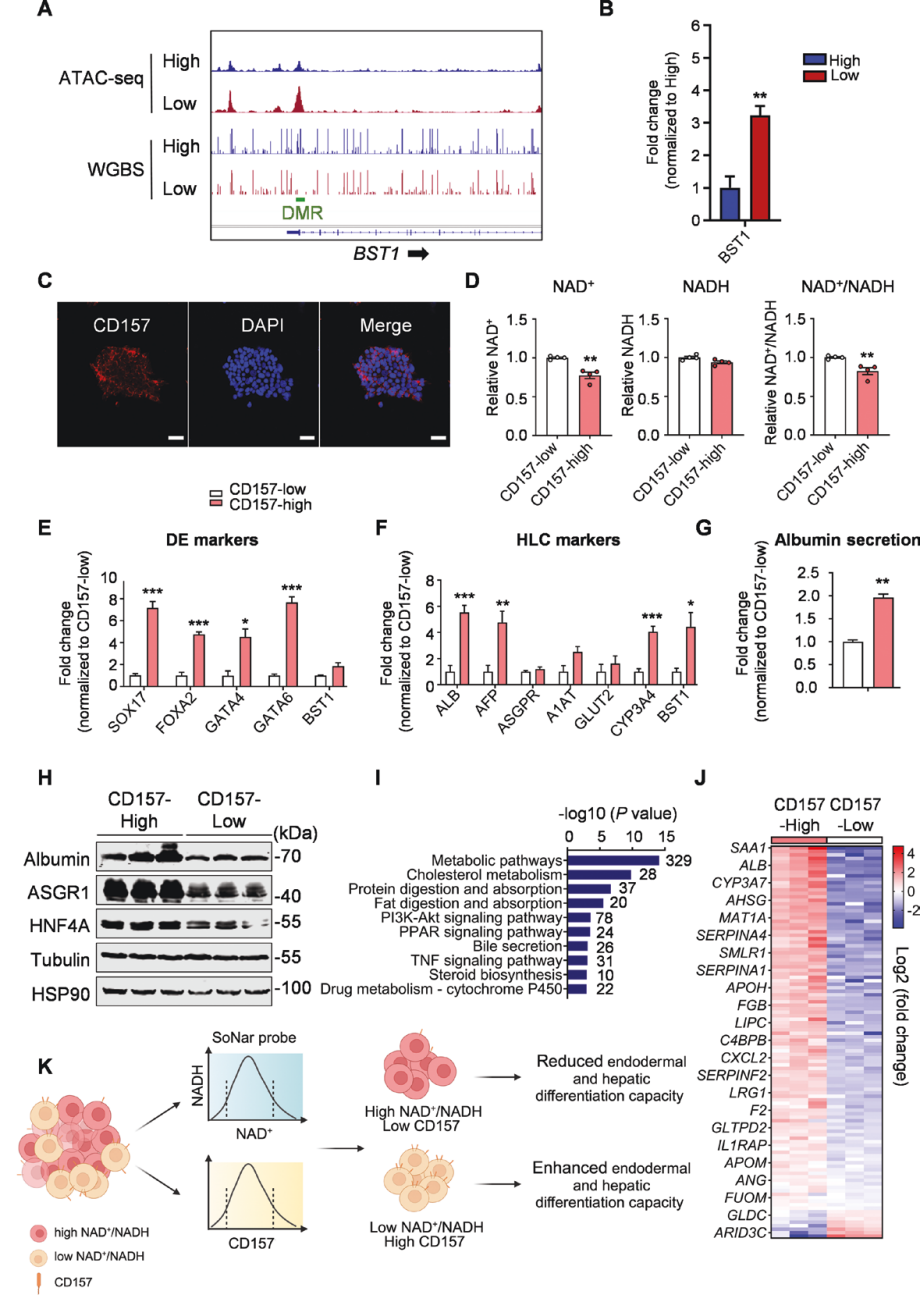
**CD157 identifies hPSCs with differentials in NAD^+^ level and hepatic differentiation capacity.**(A) ATAC-seq and WGBS signals in *BST1* gene in SoNar-High and -Low cells. (B) Expression levels of *BST1* in SoNar-High and -Low hPSCs. *n* = 3 biological replicates. (C) Representative CD157 staining of hPSCs. Scale bar, 50 μm. (D) Measurement of the NAD^+^/NADH ratio in CD157-high and -low cells, respectively. *n* = 4 biological replicates. (E) Relative mRNA levels of definitive DE marker genes in CD157-high and -low hPSCs-derived DE cells. *n* = 3 biological replicates. (F) Relative mRNA levels of hepatic marker genes in CD157-high and -low hPSCs-derived hepatocytes. *n* = 4 biological replicates. (G) Measurement of albumin secretion from CD157-high and -low hPSCs-derived hepatocytes. *n* = 4 biological replicates. (H) Protein expression analysis of hepatic markers in CD157-high and -low hPSCs-derived hepatocytes. (I) Pathway analysis of differentially expressed genes between CD157-high and -low hPSCs-derived hepatocytes. (J) Heatmap presentation of differentially expressed liver-enriched genes in CD157-high and -low hPSCs-derived hepatocytes. (K) Graphical summary. This cartoon was created by Biorender. The results are presented as means ± SEM; *P < 0.05; **P < 0.01; ***P < 0.001. Two-tailed Student’s *t* test was used for two-group comparisons, and one-way ANOVA was used for multi-group comparisons

In summary, our study revealed the presence of heterogeneity in NAD metabolism within hPSCs, which can be attributed, at least in part, to intrinsic epigenetic variations. We identified CD157 expression as a potential indicator of this NAD heterogeneity. Furthermore, we discovered that hPSCs with a low ratio of NAD^+^/NADH or high CD157 expression exhibit an augmented capacity for endoderm and hepatic differentiation (Fig. 2K). These findings provide a novel avenue for reducing cellular heterogeneity and improving the translational potential of hPSCs in future applications.

## Research limitations

While the phenomenon observed in this study is evident in the hPSC lines used in our experiments, generalizing it to other hPSC lines requires further evaluation. It is essential to investigate whether the level of heterogeneity in cellular NAD metabolism and CD157 surface expression varies across different hPSC lines. In addition, it would be valuable to explore whether these variations persist within the same hPSC line at different passage numbers or under different culture conditions and whether new heterogeneity arises in sorted-out subpopulations after subsequent long-time culture. In summary, further investigations are needed to assess the broader applicability and stability of these findings across different hPSC lines and experimental conditions.

## Supplementary Material

lnae026_suppl_Supplementary_Material
