# Supplementary material for: CD157 selectively identifies hPSCs with enhanced hepatic differentiation capacity
Source: Life Med. 2024 Jun 5;3(4):lnae026. doi: 10.1093/lifemedi/lnae026 (PMC11749559; doi:10.1093/lifemedi/lnae026)
Supplement: lnae026_suppl_Supplementary_Material [file lnae026_suppl_Supplementary_Material.docx]

**Supplementary Information for “CD157 selectively identifies hPSCs with enhanced hepatic differentiation capacity”**

**Materials and Methods**

**Cell culture**

Human embryonic kidney 293T (HEK293T) cells were cultured in DMEM medium supplemented with 10% fetal bovine serum and 1% penicillin/streptomycin. Human pluripotent stem cells, including 1016 induced pluripotent stem cells (1016 iPSC) (obtained from the HSCI iPS Core, Harvard), H1 hESCs and H7 hESCs (obtained from Wi Cell Research Institute, Madison, WI) were cultured in feeder-free adherent culture using chemically defined mTeSR1 medium (STEMCELL Technologies, 05850) with penicillin and streptomycin. The plates were precoated with Geltrex matrix (Invitrogen, A1413202). The cells were regularly passaged using Accutase (Invitrogen, A1110501) every 4–5 days.

**Plasmids**

The SoNar plasmid was obtained from Professor Yang Yi ^1^. The MitoTimer (52659)^2^, Grx (64990)^3^, and Trx (98997) ^4^ plasmids were purchased from addgene with plasmid backbone replaced with the pCDH vector (System Biosciences, CD527A-1) for lentivirus packaging. The plasmids of *ART3*-Flag and *BST1-*Flag were cloned into the pcDNA3.0 vector with a FLAG-tag at 3' of the cDNA, respectively.

**Lentivirus packaging**

To prepare lentiviruses, HEK293T cells in each 10-cm dish were transfected with 10 μg SoNar or other core plasmids together with 6.5 μg pMDL, 2.5 μg pRev and 3.5 μg pVSVG packaging plasmids. After transfection for 48 and 72 h, the virus-containing supernatants were collected and centrifuged at 20,000 rpm for 2 h at 4°C to pellet the viral particles. The viral pellets were then resuspended in DMEM at 4°C overnight.

**Differentiation of 1016 iPSC into DE and HLCs**

Differentiation of 1016 iPSC into DE and HLCs were following a similar protocol as described^5^. Briefly, 1016 iPSC were 1) incubated in RPMI-B27 (RPMI-1640 from Invitrogen, 11875093; B27 supplement from Invitrogen, 12587010) medium supplemented with recombinant activin A (100 ng/mL, PeproTech, AF-120-14E) and LY-294002 (5 μM, Selleck, S1105) for 3 days to obtain definitive endoderm; 2) RPMI-B27 supplemented with BMP4 (20 ng/mL, PeproTech, 120-05) and FGF2 (5 ng/mL, PeproTech, AF-100-18B) and 0.5% DMSO for 5 days to get hepatoblasts; 3) RPMI-B27 supplemented with HGF (20 ng/mL, PeproTech, 100-39) and 0.5% DMSO for 5 days to get immature hepatocytes; and 4) HCM Hepatocyte Culture Medium (Lonza, CC-3198) supplemented with HGF (20 ng/mL), Oncostatin M (20 ng/mL, PeproTech, 300-10), dexamethasone (100 nM, Sigma, D4902) and 0.5% DMSO for 5 days to get mature HLCs.

**Directed differentiation toward ectoderm and mesoderm**

Directed differentiation of 1016 iPSCs into ectoderm or mesoderm was performed using the STEMdiff Neural Induction Medium (Stemcell, 05835) or STEMdiff Mesoderm Induction Medium (Stemcell, 05220), respectively, according to the manufacturer’s manual.

**Flow cytometry and cell sorting**

Sorting of the SoNar-high and SoNar-low cells was performed in the SoNar-infected 1016 iPSCs under the condition of excitation light at 405 nm/488 nm and emission light at 525 nm, with a total 5 rounds of enrichment. For enrichment of CD157-high and CD157-low cells, 1016 iPSCs were stained with a PE-conjugated CD157 antibody (ThermoFisher, 12-1579-42, 1:100) at 4°C for 30 min, followed by sorting with flow cytometry by CD157 expression level. This sorting process was also repeated for a total of 5 rounds to obtain the population of cells with high and low CD157 expression, respectively.

**Measurement of the intracellular NAD^+^ and NAD^+^/NADH ratio**

The determination of NAD^+^, NADH, and NAD^+^/NADH ratio was performed using the detection methods provided by the NAD^+^/NADH assay kit (Sigma, MAK037-1KT). A total of 5 × 10^5^ cells were harvested and sorted directly into 400 μL of the NAD^+^/NADH extraction buffer. The optical density at OD 450 nm was then measured using a multi-well spectrophotometer. The final NAD^+^ and NADH content, as well as the NAD^+^/NADH ratio, in each sample were calculated based on the standard curve generated from NADH standards provided in the kit. Data were normalized to protein content.

**Generation of CRISPR-Cas9 knockout cell lines**

The 20 bp sequence of sgRNA targeting individual genes was inserted to lentiCRISPR v2 plasmid and used for lentivirus packaging. The target sequences used are 5'-CCTGAAATGTACGGACAGGA-3' for *ART3-sgRNA1*, 5'-ATACGTTCCCCAACTGCTAA-3' for *ART3-sgRNA2*, 5'-CCAGCAATTAGATACTGTGT-3' for *ART3-sgRNA3*; 5'-CCCAGGGGTGCGCGGCATCG-3' for *BST1-sgRNA1*, 5'- GGGCCGCCATCGGGGAACTT-3' for *BST1-sgRNA2*, 5'- GGACCAAAGTTCCCCGATGG-3' for *BST1-sgRNA3*, 5'- GTTGCTGCTGGCGGCGGGCG-3' for *BST1-sgRNA4.* Lentiviruses carrying CRISPR-Cas9 targeting individual genes or empty lentiCRISPR v2 vector as control viruses were packaged. The 1016 iPSC were infected with control and CRISPR lentiviruses and selected with puromycin (1 μg/mL). Cells were next subjected to genomic DNA extraction for T7EI analysis (NEB, E3321) to determine gene editing efficiency. Primers used in T7EI analysis are 5'-CAGGACTGCCACCCATTTCT-3' and 5'-ATTGGCCAGCCATCTTCACA-3' for *ART3*, 5'-GCGAGATATCCGAGCGAGAG-3' and 5'-CCCCTCACCCCGAACTTTAG-3' for *BST1*.

**Fluorescence microscopy**For fluorescence microscopy, SoNar-, Grx-, Trx-, SoNar-high and low, CD157-high and low-expressing cells were plated on a 35 mm glass-bottom dish coated with Geltrex matrix in mTeSR1. Images were acquired using Zeiss (LSM880NLO FLIM) microscope.

**Colony formation**

SoNar-high and -low cells were isolated and seeded at a density of 1 × 10^4^ cells per well in a 12-well plate. The cells were cultured for 7 days with medium changed every other day. The supernatant was then removed and the cells were washed once with PBS before being fixed with 4% PFA for 10 min. After fixation, the cells were washed three times with distilled water and then stained with crystal violet (Beyotime, C0121) for 10 min. The cells were observed and imaged after thorough washing with distilled water.

**RNA-seq analysis**

RNA purification, reverse transcription, library construction and sequencing were performed at Shanghai Majorbio Bio-pharm Biotechnology Co., Ltd. (Shanghai, China) according to the manufacturer’s instructions (Illumina, San Diego, CA). For data analysis, the raw RNA-seq reads were aligned to human genome (hg38) by STAR. The number of reads for each RefSeq gene was counted by HTSeq. Differentially expressed genes were then identified by DEseq2 by using log_2_-fold change ≥ 1.5 and *p*Value < 0.05 as cutoff. GO term and pathway annotations were downloaded from Gene Ontology website. GO enrichment analysis was performed by homemade scripts of R scripts. The 39 transcription factor gene sets involved in regulating pluripotent stem cells’ stemness were retrieved from the Stemchecker website; the 263 liver-enriched gene sets were retrieved from the Human Protein Atlas project.

**ATAC-seq analysis**

Cells were washed twice with PBS and resuspended in the homogenization buffer (10 mM Tris-HCl, pH 7.4, 10 mM NaCl, 0.1% IGEPAL CA-630 and 3 mM MgCl_2_) for 10 min on ice to isolate the nuclei. The suspension of nuclei was spun at 500 *g* at 4°C for 5 min to remove the supernatant. Nuclei were then incubated with Tn5 transposition reaction mix at 37°C for 30 min. The stop buffer was then added directly into the reaction to end the tagmentation. DNA was purified using the MolPure® PCR Purification Kit (Yeasen, 19106ES70) and purified with VAHTS DNA Clean Beads (Vazyme, N411). The ATAC-seq library was established using TruePrep DNA Library Prep Kit V2 for Illumina (Vazyme, TD502) as described. Then, the qualified libraries were sequenced on Illumina Nova Seq 6000.

FASTQ files were checked with Fastqc (v. 0.11.9), and adapters were removed from the raw reads with Cutadapt (v. 1.18). The alignment SAM files and remove duplicate reads were sorted using Samtools (v. 1.3.1). Peak-calling was on nucleosome-free reads performed using MACS2 (v. 2.1.1.20160309). The peak annotation was realized with HOMER (v. 4.1.5).

**Whole genome bisulfite sequencing (WGBS-seq) analysis**

The WGBS-seq was performed in GENEWIZ, INC, SuZhou. Brifely, the library preparations were constructed following the manufacturer’s protocol. For each sample, 1 μg genomic DNA was randomly fragmented to < 500 bp by sonication. Size selection of adaptor-ligated DNA was then performed using beads, and fragments of about 410 bp were recovered. Then bisulfite conversion was performed using the related Kit. Each sample was then amplified by PCR for 10 cycles using P5 and P7 primers. The PCR products were cleaned up using beads, validated using an Qsep100 (Bioptic, Taiwan, China), and quantified by Qubit3.0 Fluorometer (Invitrogen, Carlsbad, CA, USA).

Then libraries with different indices were multiplexed and loaded on an Illumina instrument according to manufacturer’s instructions (Illumina, San Diego, CA, USA). Sequencing was carried out using a 2 × 150 paired-end configuration; image analysis and base calling were conducted by the Illumina pipeline (image control software (HCS/MCS) + OLB + GAPipeline-1.6). Using Cutadapt (V1.9.1) removed the sequences of adaptors. Bismark (V0.7.12) was used to map clean data to reference genome, then call methylated cytosine (mC) by coverage 2 cytosine command. Differentially methylated cytosines between the treatment and the control group were detected by methylKit (V0.9.5). swDMR (V1.0.7) was used to reveal set of differentially methylated regions (DMR). Enrichment for target genes was completed with GO/KEGG database.

**ELISAs, immunocytochemistry and western blot analysis**

For ELISA experiments, supernatant from differentiated HLCs from CD157-high and -low hPSCs were collected at the end of the differentiation to determine the concentration of human albumin (Abcam, ab108788). Total protein amount was used for normalization.

For immunocytochemistry analysis, hPSC, differentiated DE or HLCs at indicated days were fixed, permeabilized with 0.1% Triton-X-100, and counterstained for CD157 (ThermoFisher, 25-1579-41), FOXA2 (Abcam, ab108422) as indicated. Nuclei were visualized with DAPI stain (Sigma, D8427) and images were taken by microscopy (OLYMPUS, IX73).

For Western blot analysis of SoNar-High and -Low cells in different stages during DE differentiation, total proteins were extracted and subjected to regular Western blot procedure against SOX17 (Abcam, ab224637), FOXA2 (Abcam, ab108422), Albumin (Abcam, ab207327), ASGR1 (Proteintech, 11739-1-AP), HNF4A (Abcam, ab181604), Hsp90α/β (Abclonal, A5027), H3K9ac (Abcam, ab10812), H3K27ac (Abcam, ab4729), H3 (Abcam, ab10799), and Alpha Tubulin (Proteintech, 66031-1-Ig).

**ChIP-qPCR**

ChIP-qPCR assays were carried out using the SimpleChIP® Enzymatic ChIP Kit (Cell Signaling Technology, 9002) following the manufacturer’s protocol. Briefly, SoNar-High and -Low cells-derived DEs (2 × 10^7^cells) were fixed with 1% formaldehyde for 10 min at room temperature and quenched with glycine for 5 min. The cells were then lysed with the lysis buffer provided. Chromatin was later digested at 37°C for 10 min to generate fragments with an average length of 150–900 bp. The samples were then sonicated on ice and supernatant was transferred into a new tube. 2% supernatant was saved for input at this stage, and the remnant was subjected to immunoprecipitation at 4°C overnight with the following antibodies, respectively: anti-H3K9ac (Abcam, ab10799) and IgG (Cell Signaling Technology, 2729) as control. Each reaction was then incubated with protein G agarose beads for another 2 h at 4°C. After standard washes, elution buffer was added to all immunoprecipitation samples and the input samples. DNA from each sample was then purified for real-time PCR. Primers used in the real-time PCR were as following: 5'-TTGGCCACATCTGTGCAGAAAA-3', and 5'-GTCGCGGTCTGGTCTACAGC-3' for *SOX17*; 5'-ACTCTAGGAGGCGTCATCCA-3', and 5’-TCCCAGTCTCTCCGAAAACG-3' for *FOXA2*. Fold enrichment was then calculated as: Percent input = 2% × 2^(C[^*^T^*^] 2% input sample – C[^*^T^*^] IP sample)^; C[*T*] = CT = threshold cycle of PCR reaction.

**Quantitative RT-PCR**

Total RNA extraction, reverse transcription and real-time PCR were performed using standard methods. The sequences of primers were as following: 5'-GCCGCCAGCTCACCAT-3' and 5'-TCGATGGGGTACTTCAGGGT-3' for *ACTB* gene; 5'-TTCGTGTGCAAGCCTGAGAT-3' and 5'-TAATATACCGCGGAGCTGGC-3' for *SOX17* gene; 5'-TTTTAAACTGCCATGCACTCG-3' and 5'-TTCATGTTGCTCACGGAGG-3' for *FOXA2* gene; 5'-CGACACCCCAATCTCGATATGTT-3' and 5'-ACAGATAGTGACCCGTCCCA-3' for *GATA4* gene; 5'-AGCGCGTGCCTTCATCA-3' and 5'-GTGGTAGTTGTGGTGTGACAGTTG-3' for *GATA6* gene; 5'-GCACACTTTCTGAGAAGGAGAG-3' and 5'-CACTTCTCTACAAAAGCTGCG-3' for *ALB* gene; 5'-TCAGTGAGGACAAACTATTGGC-3' and 5'-GGGTTTACTGGAGTCATTTCATG-3' for *AFP* gene; 5'-GGAACCTATGATCTGAAGAGCG-3' and 5'-TGGTCAGCACAGCCTTATG-3' for *A1AT* gene; 5'-GAGCAGAAATTTGTCCAGCAC-3' and 5'-CCTCCAGTTCTTGAAGCCC-3' for *ASGPR* gene; 5'-TTCACCGTGACCCAAAGTAC-3' and 5'-TGAGAGCAAACCTCATGCC-3' for *CYP3A4* gene; 5'-TTTCAGTCAAGGACCACGTC-3' and 5'-GAGCACTCCAGCAAAGAGG-3' for *GLUT2* gene; 5'-CGGCTACCACATCCAAGGAA-3' and 5'- GCTGGAATTACCGCGGCT-3' for 18S RNA. 5'-ACTTGCGGGACATCTTCCTG-3' and 5'-AAAGGTCATAGTCTGAGGGCA-3' for *BST1* gene; 5'-ACATCAAAGCTCTGCAGAAAGAACT-3' and 5'-CTGAATACCTTCCCAAATAGAACCC-3' for *POU5F1* gene; 5'-GAATGAAATCTAAGAGGTGGCA-3' and 5'-CCTGGTGGTAGGAAGAGTAAAGG-3' for *NANOG* gene; 5'-CCCAAGTGACAAGGGACAAC-3' and 5'-CAGTCTGCCAAGGAACCACT-3' for *MESP1* gene; 5'-CAAGAACGGCAGGAGGATGT-3' and 5'-AAGGAGTACATGGCGTTGGG-3' for *TBXT* gene; 5'-GCCGACAATAACATGCAGGG-3' and 5'-GTGGGAACCAGTATTAGGAGACT-3' for *EOMES* gene; 5'-CCGAGTCCAGGATCCAGGTA-3' and 5'-CTCTGACGCCGAGACTTGG-3' for *MIXL1* gene; 5'-AACGCGGAGAAGTGGAACAAG-3' and 5'-CTGTCCGAGTCCAAATCGC-3' for *GSC* gene; 5'-TGAAGGGCAATCACAACAGG-3' and 5'-TGACCCCAACATGACCTCTG-3' for *NESTIN* gene; 5'-CATACCAAGCGTGTCATCAATAAAC-3' and 5'-TGCGCCCATCTGTTGCT-3' for *PAX6* gene; 5'-CCCAAGCAAGTCAAGCGACA-3' and 5'-AAGCCGCTGAAGTTGAGCC-3' for *ASCL1* gene; 5'-GCAACCATGATTCTAGTGGACA-3' and 5'-CTTTAGCAGTTGGGGAACGTAT-3' for *ART3* gene; 5'-CTGGCGGCTTTGATGATCG-3' and 5'-ACAACCCACATAGGTATCGTCA-3' for *ART5* gene.

**Statistical analysis**

The unpaired, two-tailed Student’s *t* tests were used for experiments with two groups, and one-way ANOVA test and *post-hoc* Bonferronic multiple-comparison tests were used for experiments that contained more than two groups. All data are represented as means with SEM.

**Research ethics**

The DE and HLCs derived from 1016 iPSC (obtained from the HSCI iPS Core, Harvard) via direct *in vitro* differentiation and gene editing established by previous study^5^. Directed differentiation of 1016 iPSC into ectoderm or mesoderm was performed using the STEMdiff Neural Induction Medium or STEMdiff Mesoderm Induction Medium, respectively, according to the manufacturer’s manual. There are no relevant ethical issues.

**Data availability**

The data supporting the findings of this study are available within the article and its supplementary materials.

**References**

1. Zhao, Y.*, et al.* SoNar, a Highly Responsive NAD+/NADH Sensor, Allows High-Throughput Metabolic Screening of Anti-tumor Agents. *Cell Metab* **21**, 777–789 (2015).

2. Laker, R.C.*, et al.* A novel MitoTimer reporter gene for mitochondrial content, structure, stress, and damage in vivo. *J Biol Chem* **289**, 12005–12015 (2014).

3. Gutscher, M.*, et al.* Real-time imaging of the intracellular glutathione redox potential. *Nat Methods* **5**, 553–559 (2008).

4. Fan, Y., Makar, M., Wang, M.X. & Ai, H.W. Monitoring thioredoxin redox with a genetically encoded red fluorescent biosensor. *Nat Chem Biol* **13**, 1045–1052 (2017).

5. Li, S.*, et al.* Genetic and Chemical Screenings Identify HDAC3 as a Key Regulator in Hepatic Differentiation of Human Pluripotent Stem Cells. *Stem Cell Reports* **11**, 22–31 (2018).


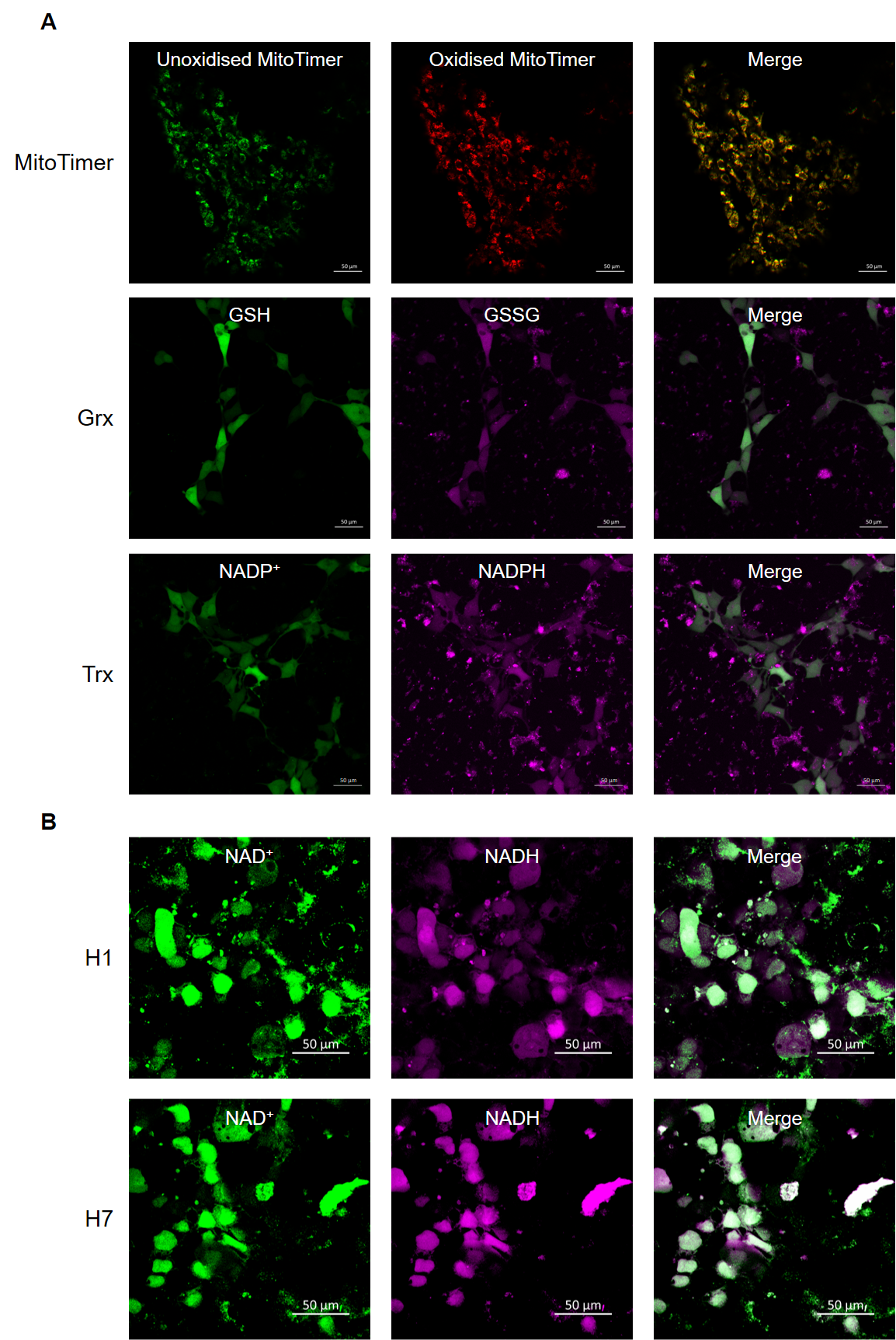


**Figure S1. Assessment of the metabolic heterogeneity in hPSCs with genetic probes.**

(A) Representative images for the MitoTimer, Grx, and Trx fluorescence in 1016 hPSCs infected with lentivirus carrying individual genetic probes, respectively. Scale bar, 50 µm.

(B) Representative images for the NAD^+^/NADH fluorescence in H1 and H7 hPSCs infected with SoNar lentivirus. Scale bar, 50 µm.

**
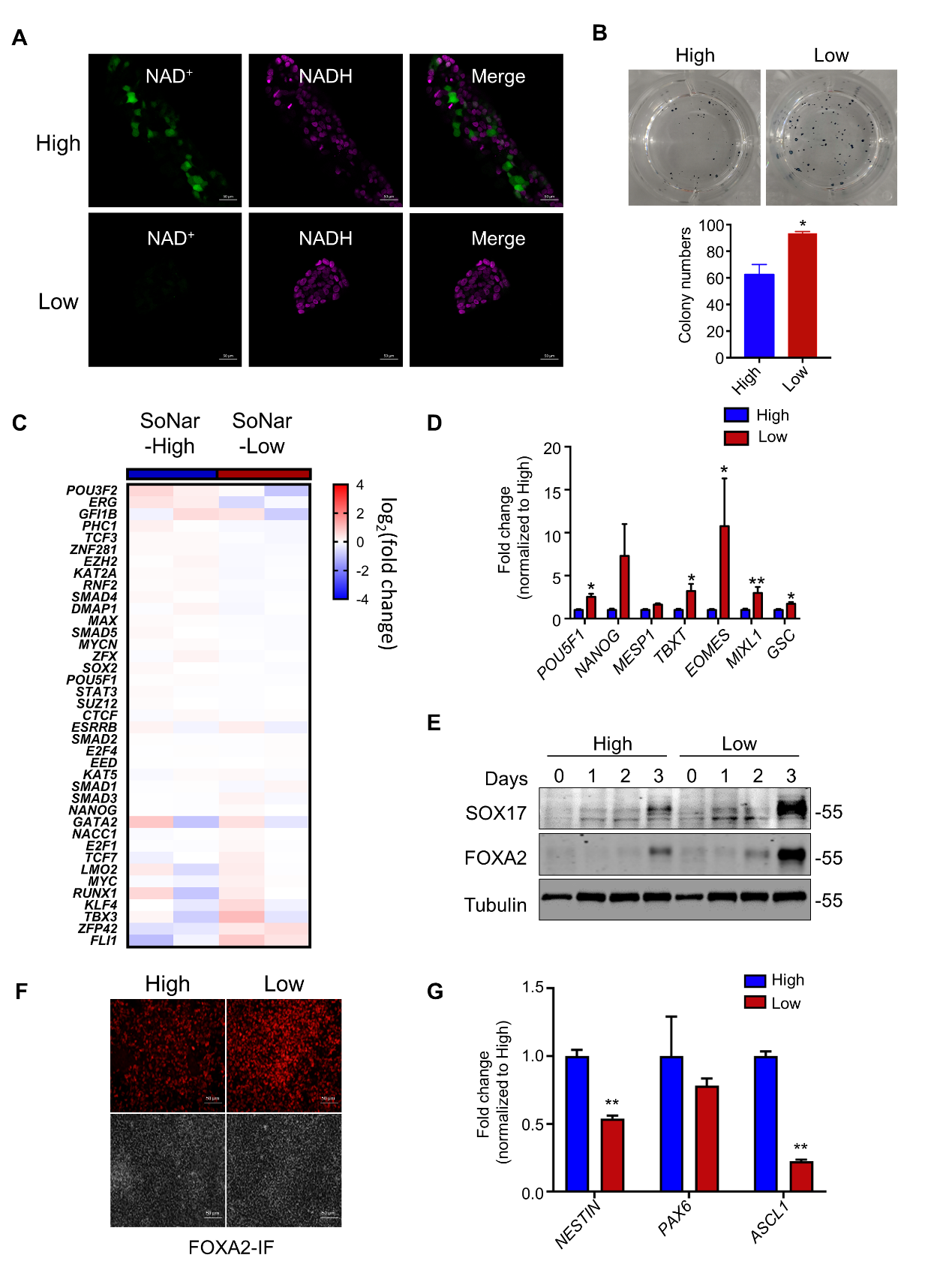
**

**Figure S2. The cellular heterogeneity of NAD^+^ level in hPSCs affects self-renewal and pluripotency.**

(A) Representative images for the NAD^+^/NADH fluorescence in SoNar-High (High) and SoNar-Low (Low) hPSCs. Scale bar, 50 µm.

(B) Evaluation of colony number formation capacity of the SoNar-High and SoNar-Low hPSCs. *n* = 3 biological replicates.

(C) Heatmap presentation of expression levels of the transcription factor sets involved in regulating pluripotent stem cell stemness in SoNar-High (High) and SoNar-Low (Low) hPSCs.

(D**)** Relative mRNA levels of mesoderm marker genes in SoNar-High and -Low cells upon mesoderm differentiation. *n* = 3 biological replicates.

(E) Western analysis of endoderm marker SOX17 and FOXA2 in SoNar-High and -Low cells along endoderm differentiation.

(F) Immunofluorescence of endoderm marker FOXA2 in SoNar-High and -Low cells upon endoderm differentiation.

(G) Relative mRNA levels of ectoderm marker genes in SoNar-High and -Low cells upon ectoderm differentiation. *n* = 3 biological replicates.

The results are presented as means ± SEM; *, *p* < 0.05; **, *p* < 0.01. Two-tailed Student’s *t* test was used for two-group comparisons, and one-way ANOVA was used for multi-group comparisons.

**
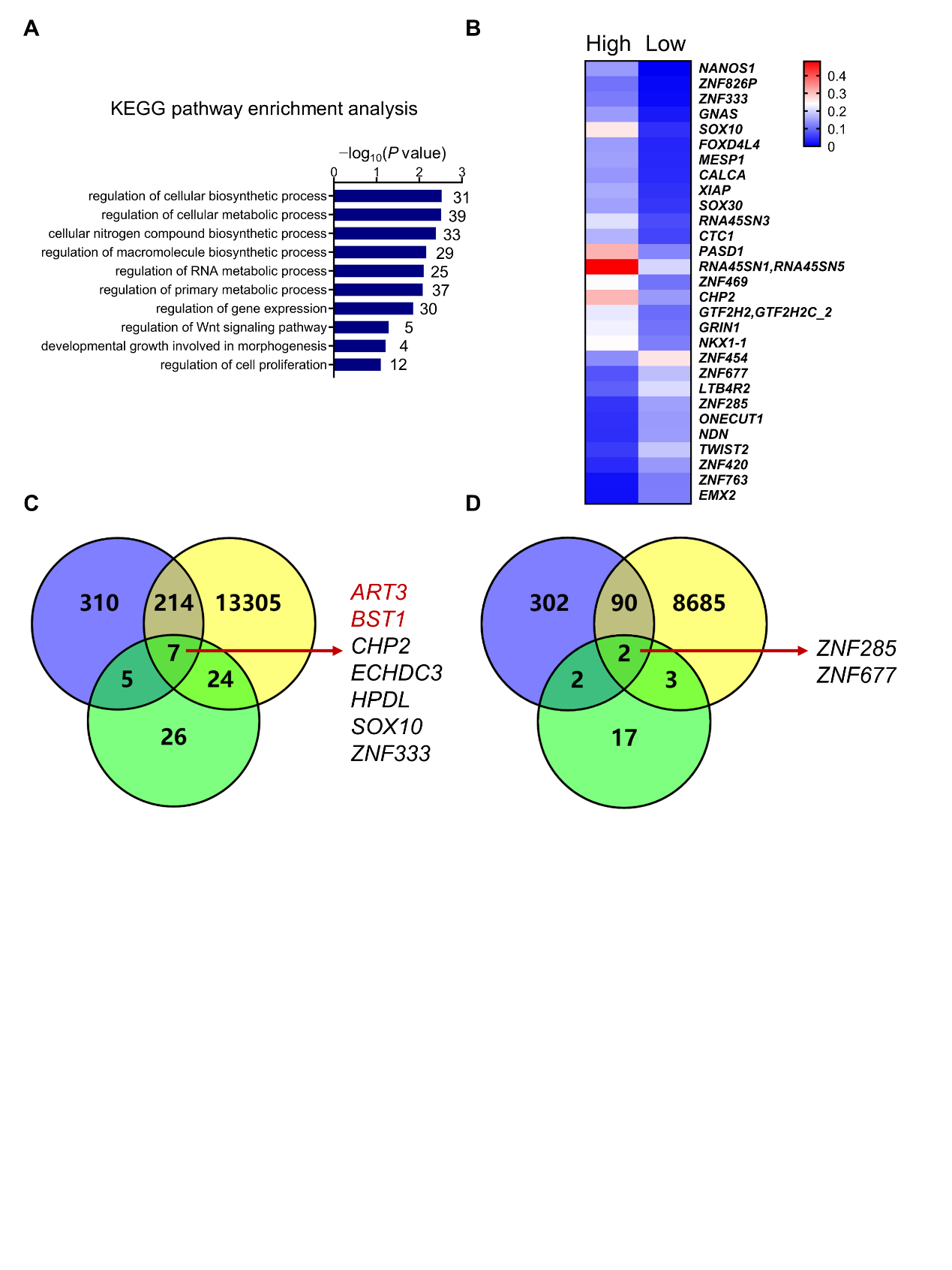
**

**Figure S3. DNA methylation analysis of the SoNar-High and -Low hPSCs.**

(A) KEGG analysis of genes showing differential signals in the WGBS-seq in SoNar-High and -Low hPSCs.

(B) Representative genes involved in the cellular biosynthetic process as in (A).

(C) Overlap analysis of genes that show increased signals in the RNA-seq and ATAC-seq, and decreased signals in the WGBS-seq.

(D) Overlap analysis of genes that show decreased signals in the RNA-seq and ATAC-seq, and increased signals in the WGBS-seq.

**
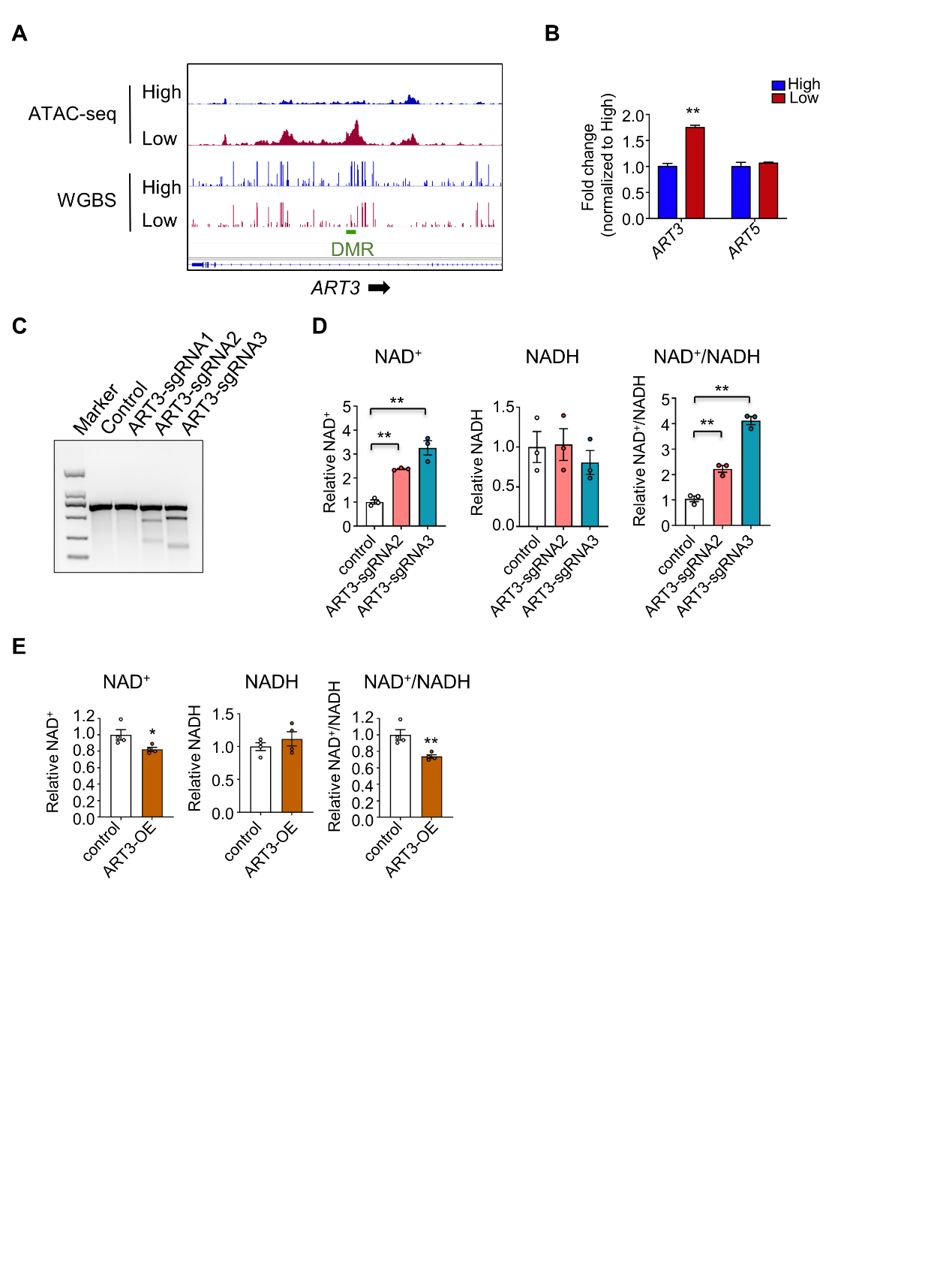
**

**Figure S4. ART3 expression affects the NAD^+^ level in hPSCs.**

(A) ATAC-seq and WGBS signals in *ART3* gene in SoNar-High and -Low cells.

(B) Expression levels of *ART3* and *ART5* in SoNar-High and -Low hPSCs. *n* = 3 biological replicates.

(C) T7E1 analysis of hPSCs treated with CRISPR-*ART3* or control viruses.

(D) Measurement of cellular NAD^+^ and NAD^+^/NADH ratio in hPSCs treated with CRISPR-*ART3* or control viruses. *n* = 3 biological replicates.

(E) Measurement of cellular NAD^+^ and NAD^+^/NADH ratio in hPSCs treated with ART3 overexpression (ART3-OE) or control viruses. *n* = 4 biological replicates.

The results are presented as means ± SEM, *, *p* < 0.05; **, *p* < 0.01. Two-tailed Student’s *t* test was used for two-group comparisons, and one-way ANOVA was used for multi-group comparisons.


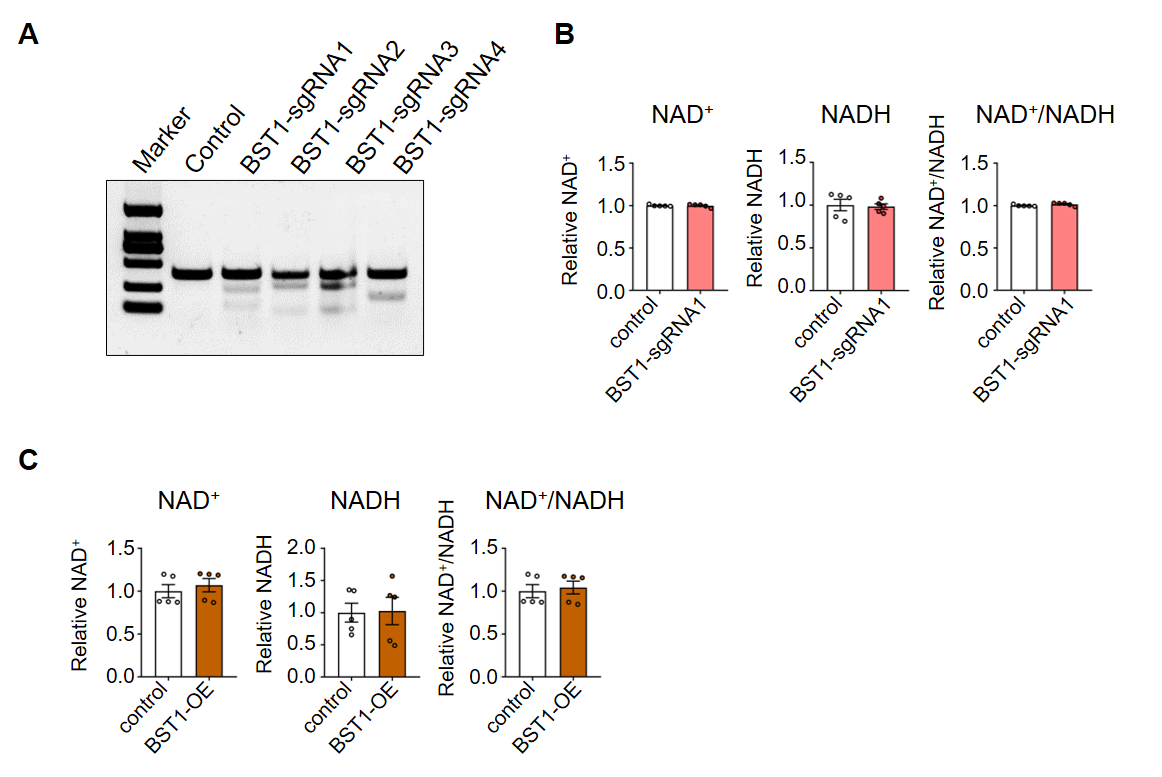


**Figure S5. BST1 expression does not regulate the NAD^+^ level in hPSCs.**

(A) T7E1 analysis of hPSCs treated with CRISPR-*BST1* or control viruses.

(B) Measurement of cellular NAD^+^ and NAD^+^/NADH ratio in hPSCs treated with CRISPR-*BST1* or control viruses. *n* = 5 biological replicates.

(C) Measurement of cellular NAD^+^ and NAD^+^/NADH ratio in hPSCs treated with BST1 overexpression (BST1-OE) or control viruses. *n* = 5 biological replicates.

The results are presented as means ± SEM, *, *p* < 0.05; **, *p* < 0.01. Two-tailed Student’s *t* test was used for two-group comparisons, and one-way ANOVA was used for multi-group comparisons.
